# Correction of the curve of spee using clear aligner therapy: A finite element analysis of three lower anterior intrusion protocols

**DOI:** 10.1371/journal.pone.0341447

**Published:** 2026-01-27

**Authors:** José Alejandro Guerrero-Vargas, Carina Cristina Montalvany-Antonucci, Sandra Melisa Velez-Muriel, Natália Couto Figueiredo, Soraia Macari

**Affiliations:** 1 School of Sciences and Engineering, Universidad del Rosario, Bogotá, Colombia; 2 Department of Restorative Dentistry, Faculty of Dentistry, Federal University of Minas Gerais, Belo Horizonte, Minas Gerais, Brazil; University of Zurich, SWITZERLAND

## Abstract

**Background:**

Correction of the curve of Spee (COS) often requires lower anterior intrusion, which remains one of the least accurate tooth movements in clear aligner therapy (CAT). This limited accuracy may be influenced by the anchorage system or the design of the intrusion strategy. This study aimed to evaluate the movement trends and stress distribution in the lower anterior teeth subjected to three different intrusion protocols using finite element analysis (FEA).

**Methods:**

Three-dimensional models of the mandibular dentition, periodontal ligaments (PDLs), bone, attachments, and clear aligners were constructed using Materialise Mimics and Materialise 3-matic software. The assembly of the anatomical structures was developed using Autodesk Inventor, and FEA was performed using FeBio software. Three protocols with different anterior intrusion designs were evaluated: S1 (simulation 1) — intrusion displacements of 0.25 mm were applied simultaneously to the canines, central, and lateral incisors; S2 (simulation 2) — intrusion displacements of 0.25 mm were applied only to the lateral and central incisors; S3 (simulation 3) — intrusion displacements of 0.25 mm were applied only to the canines. Total displacement, equivalent strain, and the distribution of minimum and maximum principal stresses were analyzed.

**Results:**

Simultaneous intrusion (S1) produced the most balanced movement with the lowest stress in the target teeth and controlled bone displacement. Intruding only the incisors (S2) increased PDL stress and anchorage extrusion but also caused minor canine intrusion, indicating force propagation through the aligner. Canine-only intrusion (S3) elevated stress in the canines and produced slight incisor intrusion. Posterior teeth functioned effectively as anchorage in all simulations.

**Conclusion:**

Simultaneous intrusion of canines and incisors is the most biomechanically efficient approach for COS correction with aligners, minimizing stress and unwanted side effects. Isolated intrusion of either group requires careful planning to manage secondary movements and anchorage control.

## Introduction

The curve of Spee (COS) is considered an occlusal curvature of the mandibular dentition that passes tangent to the buccal cusps of the molars up to the incisal edge when observed in the sagittal plane [1]. COS deepening has a multifactorial etiology, often associated with the eruption of permanent teeth, craniofacial growth, and malocclusion types [2]. During orthodontic treatment, one of the main goals is to level the COS, remove occlusal interferences, and achieve mandibular and muscular balance. Additionally, COS correction is essential for obtaining Class I molar and canine relationships with optimal intercuspation [3].

It is well known that COS correction involves lower anterior intrusion, posterior extrusion, or a combination of these movements [4,5]. However, the mechanics chosen can result in complications, such as counterclockwise mandibular rotation referring to the sagittal plane, which can worsen Class II malocclusion cases. Therefore, lower anterior intrusion is a key step to level the COS. Historically, different fixed appliance techniques for achieving lower anterior intrusion have been well-documented and proven clinically effective [4,6–8]. However, clear aligner therapy (CAT) has recently gained popularity due to its aesthetic appeal and patient comfort.

The evaluation of tooth movement accuracy with clear aligner therapy (CAT) began in 2009 with Kravitz et al. (2009) [9] and, was further investigated by Krieger et al. (2011) [10], Grünheid et al. (2016) [11], Charalampakis et al. (2018) [12], and Haouili et al. (2020) [13], all concluding that deep bite correction through lower incisor intrusion remains a challenging movement in CAT. This highlights the need for a better understanding of the biomechanics involved in curve of Spee (COS) leveling with aligners, especially regarding the predictability of lower anterior intrusion. In this context, Rozzi et al. (2022) [14] conducted a retrospective study comparing continuous arch-wire treatment with the Invisalign™ system, suggesting that Invisalign™ can effectively level the COS through lower incisor intrusion, although other studies report limited accuracy for this movement with CAT.

The biomechanics involved in tooth movement with CAT are complex because they depend on the material properties, production processes, attachment placement, aligner thickness, presence of additional accessories (e.g., bite ramps), fitting accuracy, patient compliance, and the designed tooth movement sequence, also known as staging [15,16]. Thus, these complexities may contribute to the low accuracy of incisor intrusion, potentially compromising the anchorage system and overall treatment outcomes [9–13].

Based on this initial premise, the biomechanical behavior involved in lower anterior intrusion with CAT when different types of intrusion protocols are designed remains a challenge [5]. Therefore, this study aimed to investigate the movement trend and stress distribution on lower anterior teeth subjected to three different intrusion designs with CAT, using finite element analysis (FEA).

## Materials and methods

Informed consent for study participation was obtained, and the use of the Cone Beam Computed Tomography (CBCT) data was approved by the Ethics Committee of Federal University of Minas Gerais under the protocol number: 47584921.6.0000.5149. All experiments were approved and performed in accordance with relevant guidelines and regulations. A single CBCT was accessed on 20/09/2021 for research purposes; it had a total of 321 tomographic images, taken every 0.25 mm.

3D models of the mandibular dentition, periodontal ligaments (PDLs), and bone were constructed using Materialise Mimics and Materialise 3-matic software (Materialise, Leuven, Belgium). Teeth and mandibular bone were reconstructed based on CBCT scans of an adult female subject with an increased curve of Spee of 3 mm depth. The COS was defined in the sagittal plane as the line passing over the incisal edges of the mandibular central incisors and the buccal cusp tips of the canines, premolars, and molars, extending to the distal cusp of the second molar. The deepest point of the COS was identified as the lowest cusp tip along this curvature, typically located between the mandibular second premolar and first molar, which represented the bottom of the occlusal curvature in the model. This geometric reference was used to characterize the initial 3-mm COS depth and ensure consistency across all simulations. PDLs were modeled as a hyper-elastic–viscoelastic film with an average thickness of 0.25 mm surrounding the roots of all teeth. In this phase, the noise was removed from the geometry, the different anatomical structures were discretized, and the models were smoothed. The results of the reconstruction are shown in Fig 1 (A-F).

**Fig 1:**
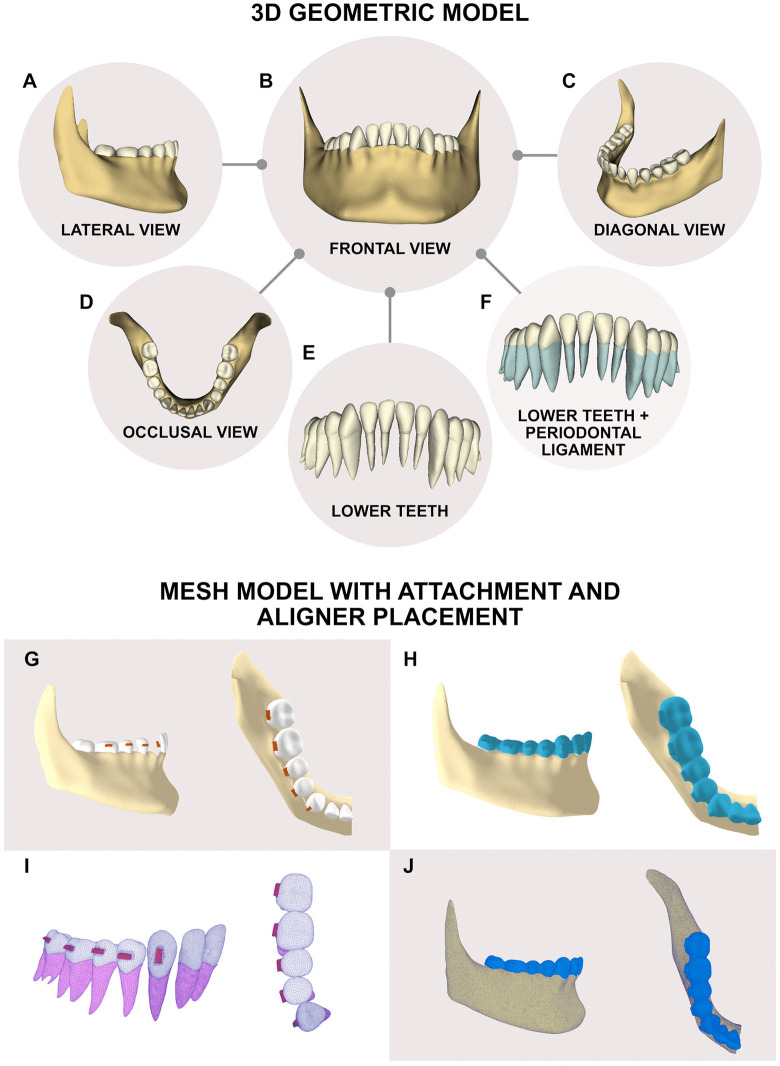
Three-dimensional geometric model. **(A)** Lateral view of the mandible; **(B)** Frontal view of the mandible; **(C)** Diagonal view of the mandible; **(D)** Occlusal view of the mandible; **(E, F)** Frontal view of the mandible, illustrating the curve of Spee and the anatomical structure of the teeth, roots, and periodontal ligament (highlighted in blue); **(G, H)** Lateral and occlusal views of the final mandibular model with attachments and the aligner (light blue cover over the teeth), respectively; **(I)** Lateral and occlusal views of the finite element mesh model of the teeth with attachments; **(J)** Lateral and occlusal views of the finite element mesh model of the complete system, including teeth, attachments, periodontal ligament, bone, and aligner.

Once reconstructed, the model was exported to Autodesk Inventor software (Autodesk, California, USA) for the assembly of the anatomical structures. Orthodontic devices (attachments) and aligners were incorporated into the model for evaluation. Molars and premolars received conventional horizontal rectangular attachments, while canine received vertical rectangular attachments to simulate the clinical condition in which they are necessary to increase aligners’ retention and anchorage for anterior intrusive movements, as previously described in the literature [17]. Horizontal rectangular attachments of 3.5 mm x 1 mm were applied to molars, and 3 mm x 1 mm to premolars. Vertical rectangular attachment of 1 mm x 3 mm was designed for the lower canine (Fig 1G, 1I). Regarding the aligners, they were designed to fit the attachments and tooth surfaces with a thickness of 0.5 mm (Fig 1H, 1J). Then, the final model was exported to the computer simulation software FeBio (FEBio Software Suite, USA) to perform the FEA. For this purpose, only half of the mandible was used, since a region of symmetry was created in the definition of the computational model. The final model is shown in Fig 2.

**Fig 2:**
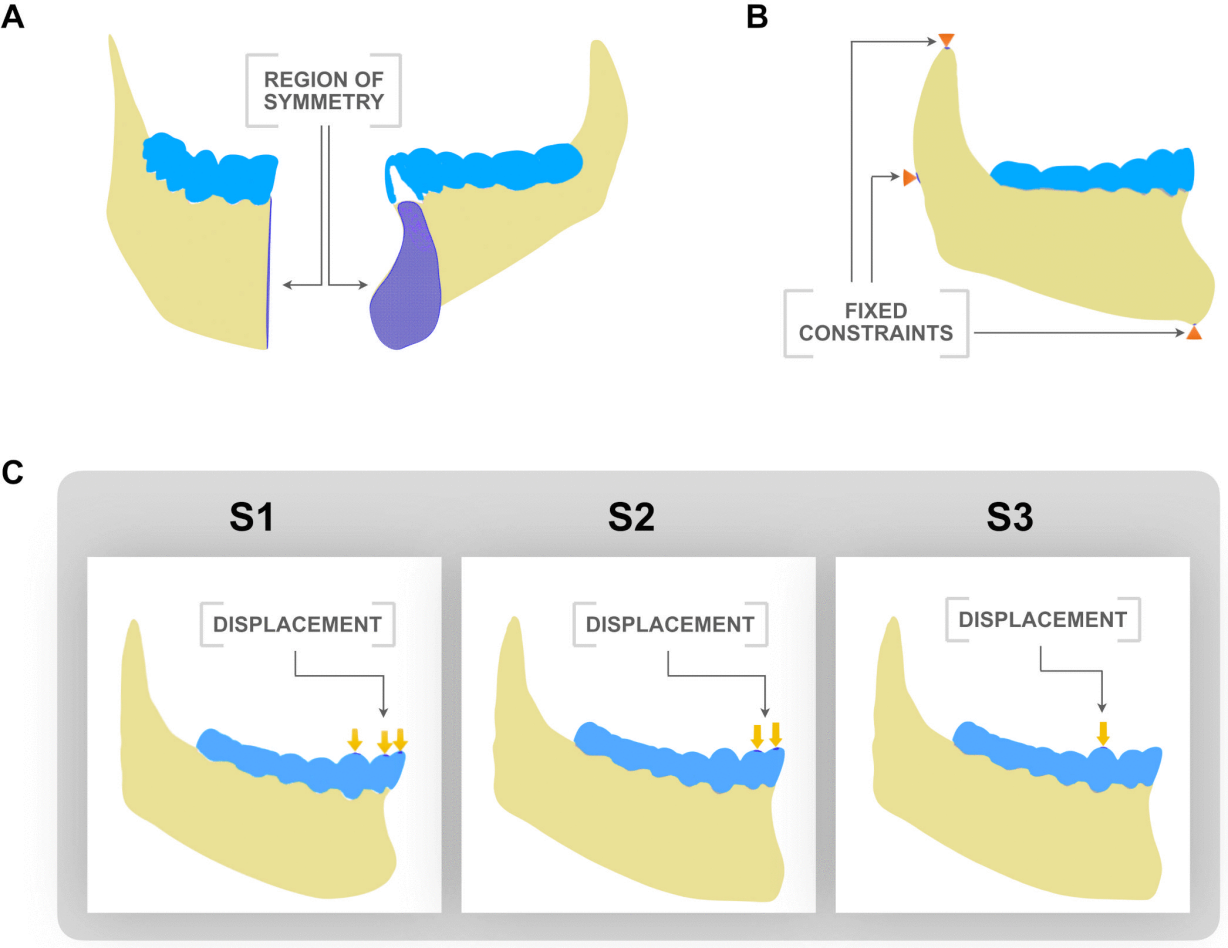
Boundary conditions and symmetry constraints applied to the finite element model. The light blue cover over the teeth represents the aligner. **(A)** The purple area represents the symmetry plane used in the simulation. **(B)** The orange arrows indicate the applied constraints: the model was restricted in the vertical direction at the superior region of the condyle and the inferior region of the mandible, and in the horizontal direction at the posterior part of the mandible. **(C)** Tooth displacement under three different intrusion designs. The yellow arrows represent the prescribed displacement applied to the aligner surface. S1: Simultaneous intrusion of canines and incisors. S2: Intrusion of only the central and lateral incisors. S3: Intrusion exclusively of the canines.

With the geometric models defined, the meshing process was conducted and the material properties of the different tissues involved were assigned. The volumetric mesh was generated using 3-matic tools, ensuring a mesh quality of at least 90% according to software criteria. The final mesh consisted of 206,370 nodes and 804,994 elements, as shown in Fig 1J, ensuring a convergence criterion of less than 5%. The materials for teeth, bone, aligner, and attachments were modeled as linear, homogeneous, and isotropic [18–21], while the PDL was defined as a hyper-elastic–viscoelastic material [17]. Material properties are summarized in Tables 1 and 2.

**Table 1:** Properties of linear materials.

Material	Modulus of Elasticity (MPa)	Poisson ratio
Tooth [18]	18600	0.31
Bone [19]	12000	0.3
Attachment [20]	12500	0.3
Aligner [21]	528	0.36

**Table 2:** Hyper-elastic–viscoelastic properties of the periodontal ligament [17].

Parameters of the second-order Ogden model	μ (MPa)	0.00554	0.11
	α	0.25	0.1153
**Relaxation function**	τ	0.13927	10,419
	G	0.0277	0.0977

Once the material properties were defined, boundary conditions were applied to simulate aligner performance under clinical conditions. Intrusion displacements of 0.25 mm were tested in three configurations (Fig 2C): *S1 (simulation 1)*, with simultaneous intrusion of the anterior teeth (central incisor, lateral incisor and canine); *S2 (simulation 2)* with intrusion applied only to the lateral and central incisors; and *S3 (simulation 3)* with intrusion applied exclusively to the canine (Table 3).

**Table 3:** Summary of intrusion configurations simulated in the study.

Simulation	Teeth involved in programmed intrusion	Displacement applied (mm)	Anchorage condition
**S1**	Simultaneous intrusion of the anterior segment (canines and incisors)	0.25	Posterior teeth included as passive anchorage elements
**S2**	Isolated incisor intrusion	0.25	Canines and posterior teeth served as passive anchorage elements
**S3**	Isolated canine intrusion	0.25	Incisors and posterior teeth served as passive anchorage elements

These displacements were applied directly to the surface of the aligner. The 0.25 mm value used in each configuration represents the programmed activation of the aligner in the digital setup and was implemented as a boundary condition to generate the initial mechanical response of the system. This displacement does not correspond to an absolute apical intrusion but to the intended deformation prescribed in the aligner design. The model was constrained vertically in the upper condyle and lower mandible, and horizontally in the posterior mandible (Fig 2B). Besides, a plane of symmetry was defined in the sagittal plane of the anterior region, assuming that the force patterns of the bilateral identical teeth were basically symmetrical (Fig 2A). Therefore, subsequent analyses were focused on the mandibular right side to evaluate stress distribution and potential movement trends. *Von Mises* equivalent strain, minimum principal stress (compressive), and maximum principal stress (tensile) were calculated to assess biomechanical behavior. *Von Misses* strain was used to represent overall strain concentration, independent of direction.

## Results

### Lower anterior teeth displacements under different anchorage conditions

In S1, central and lateral incisors exhibited the greater displacement on the buccal surfaces of the crown and the cervical third of the roots (−0.25 to −0.14 mm), with a progressive reduction toward the lingual and apical regions, reaching −0.04 mm at the most lingual/apical portion of the roots. Among the anterior teeth, the central incisor displayed the highest displacement, followed by the lateral incisor and canine. The canine exhibited a maximum displacement of −0.18 mm on its mesial crown surface, which progressively decreased towards its middle and distal portions, ranging from −0.14 to −0.07 mm (Fig 3A). The anchorage teeth showed displacement ranging from −0.07 to +0.03 mm, with a more pronounced shift observed at the distal surfaces of the first and second molars (Fig 3A). When evaluating the PDL, incisors and canines demonstrated the greatest displacement in the buccal cervical third and the least on the lingual surface. Bone displacement was more pronounced in the buccal region of the mandibular body, particularly in the premolar and canine regions, progressively decreasing toward the anterior and posterior extremities of the mandible (Fig 3A). The aligner and attachments followed a similar displacement pattern in the anterior region (−0.25 mm), with a progressive reduction towards the posterior teeth, reaching +0.03 mm at the second molars.

**Fig 3:**
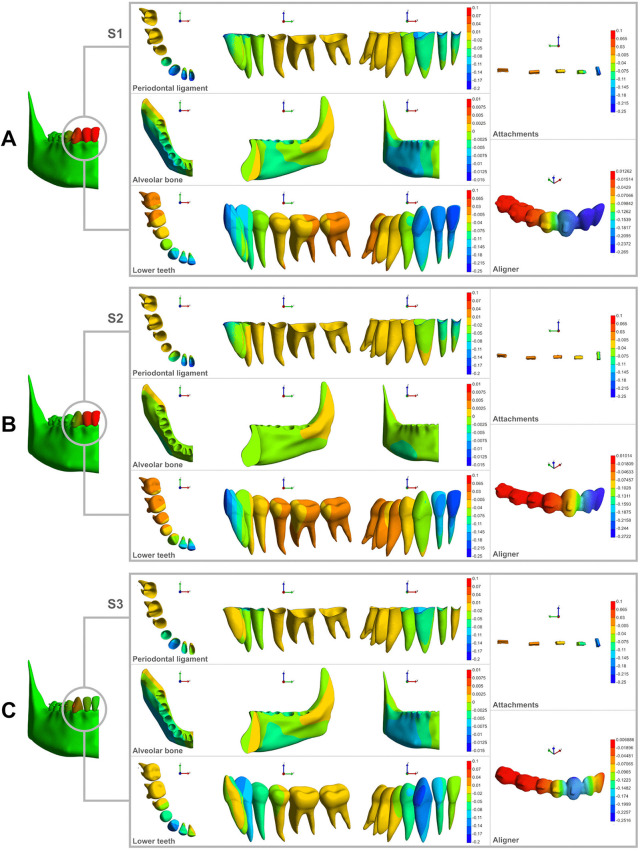
Different vertical displacement patterns (mm) observed in S1, S2, and S3 during intrusion with clear aligner therapy. **(A)** Displacement of the lower anterior and anchorage teeth when canines and incisors are intruded simultaneously. **(B)** Displacement of the lower anterior and anchorage teeth when only the incisors are intruded. **(C)** Displacement of the lower anterior and anchorage teeth when only the canines are intruded. In all cases, the first row represents PDL displacement, the second row represents alveolar bone displacement, and the third row represents tooth displacement in occlusal, lingual, and frontal views, respectively. The right column illustrates the vertical displacement of the attachments and aligner.

In S2, the greatest displacement was observed on the buccal surface of the central incisor crown (−0.25 mm), progressively decreasing towards the lingual and apical regions, reaching −0.04 mm. The lateral incisor followed a similar pattern but with a lower magnitude, exhibiting a maximum displacement of −0.18 mm at the mesial portion of the buccal crown surface. As part of the anchorage segment in this scenario, the canine displayed minimal and uniform displacement across both the crown and root, with a slight reduction towards its distal portion (−0.075 to −0.005 mm) (Fig 3B). Molars and premolars showed displacement ranging from −0.04 mm at their mesial aspects to +0.03 mm at their distal aspects, with no significant differences between the crown and root. The PDL of the incisors and canines followed the same displacement pattern observed in the corresponding teeth. In contrast, the PDL of the posterior teeth exhibited either no displacement or minimal movement. Bone displacement was minimal (−0.05 mm), localized in the buccal-inferior region of the mandible, particularly near the middle portion of the mandibular body. The aligner exhibited a maximum displacement of −0.27 mm in the incisor region, while its displacement at the canine was minimal (−0.13 mm to −0.04 mm) and nearly absent in the posterior teeth (−0.01 mm to +0.01 mm). The attachments followed a similar trend, ranging from −0.11 mm at the canine to +0.03 mm at the second molar. Overall, the incisors in S2 demonstrated a displacement pattern comparable to S1, with the lateral incisor showing a slightly lower magnitude of movement. In addition, mandibular bone displacement was notably lower in S2 compared to S1.

In S3, the canine exhibited a total displacement concentrated on the buccal/mesial surface of the crown (−0.2 mm), with a gradual reduction towards the disto-apical region (−0.08 mm) (Fig 3C). The adjacent teeth, lateral incisor and first premolar, experienced minor displacement (−0.08 to −0.11 mm), which progressively diminished toward the arch’s extremities (the midline and the most distal portion). In this scenario, the molars presented no displacement, while the second premolar and central incisor exhibited minimal displacement (−0.02 mm). The PDL of the canine displayed the greatest displacement (−0.14 mm) in the buccal cervical region, decreasing towards the lingual aspect (−0.02 mm). The PDL of the adjacent teeth showed a predominantly uniform displacement ranging from −0.05 to −0.02 mm, with an almost negligible displacement on the lingual surface of the lateral incisor. For the remaining teeth (central incisor, second premolar, and molars), PDL displacement was virtually absent. Bone displacement followed a pattern similar to that observed in S1, albeit with a slight overall reduction in magnitude. The aligner exhibited its maximum displacement of −0.25 mm in the canine region, with a progressive reduction towards both anterior and posterior regions, reaching a minimum displacement of −0.01 mm at the mesial surface of the central incisor and +0.006 mm at the molars. The displacement pattern of the attachments closely resembled that observed in S1.

### Strain and stress distribution under S1, S2 and S3 conditions

In S1, Von Mises strain distribution was primarily concentrated in the buccal and cervical regions of the PDL of the canine and incisors, gradually decreasing towards the lingual aspect of the PDL. The central incisor PDL exhibited higher strain levels compared to the other teeth (Fig 4A, 4C). Among the evaluated tooth surfaces in S1 (Fig 4B, 4D), the central and lateral incisors showed the highest strain values, particularly in the cervical and middle thirds of the buccal root surface. On the lingual surface, a slight strain concentration was observed in the cervical third, as well as on the mesial surfaces of the canine and first premolar. In the posterior region, strain values were virtually absent in both the PDL and the teeth. An increased strain concentration was detected in the attachment of the canine, while mild strain was observed in the attachments of the first premolar and second molar (Fig 4D). In S2, a strain distribution pattern similar to S1 was observed for the incisors and their respective PDLs (Fig 4A, 4B, 4E, 4F). However, in the canine root surface, strain distribution followed the same pattern as S1 but with lower intensity. The same trend was noted in the attachments of the canine and second molar. In S3, the strain distribution in the PDL and on the surfaces of the canine and first premolar was similar to that observed in S1 (Fig 4A, 4B, 4G, 4H). Interestingly, strain appeared more prominent on the mesiobuccal surface of the canine root in S1 than in S3, despite the similar overall values (Fig 4B, 4D, 4H). This likely indicates a tendency for mesiobuccal tipping of the canine in S1, while a more vertically oriented intrusive movement is suggested in S3. In this case, the highest strain concentration was located near the attachment surface.

**Fig 4:**
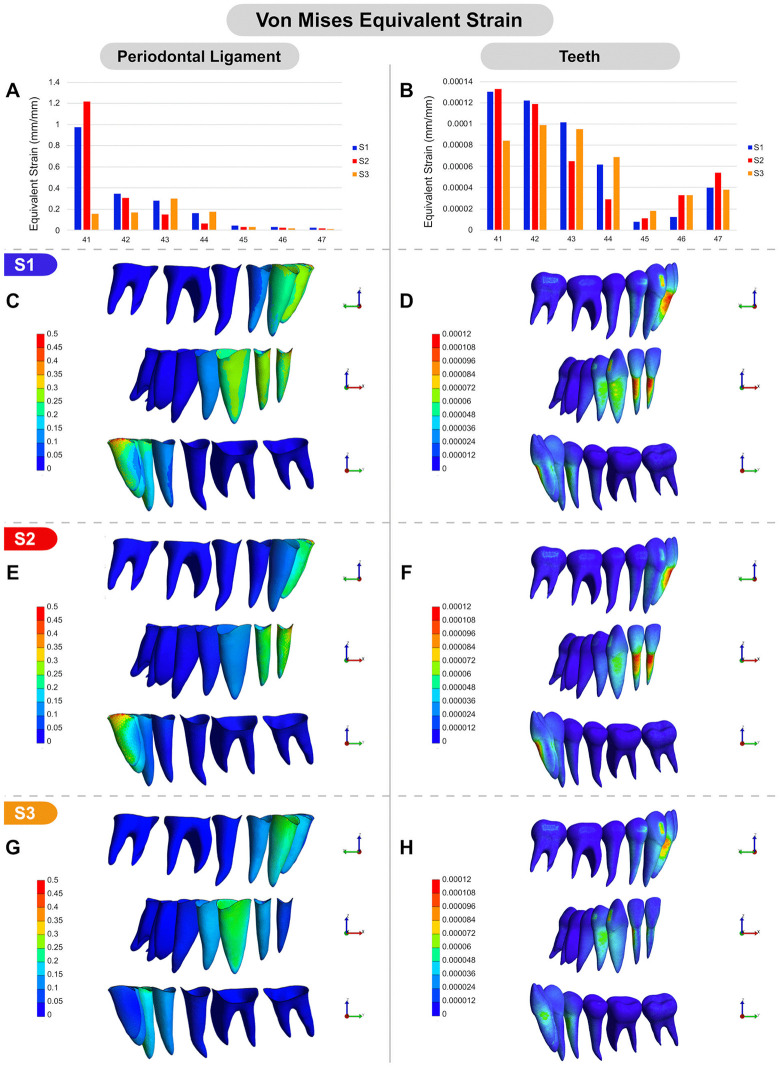
Von Mises equivalent strain distribution (mm/mm) under S1, S2, and S3 conditions. **(A, B)** Graphical comparison of von Mises equivalent strain values: (A) in the PDL of the lower teeth across S1, S2, and S3; (B) in the lower teeth across S1, S2, and S3 conditions. **(C-H)** Color-mapped visual representations of equivalent strain distribution: (C, D) under S1 in the PDL and teeth, respectively; (E, F) under S2 in the PDL and teeth, respectively; (G, H) under S3 in the PDL and teeth, respectively. All images (C-H) include buccal, frontal, and lingual views.

Additionally, in S3 simulation, the von Mises strain distribution in the incisors followed a distinct pattern. Instead of being concentrated in the buccocervical region of the incisor roots, strain was predominantly observed on the proximal root surfaces, particularly in the distocervical region. Overall, the molars and second premolars exhibited minimal strain in both the teeth and the PDL across all simulations, indicating that force transmission was primarily concentrated in the anterior region. Moreover, strain values in the teeth were consistently lower than those observed in the PDL.

Regarding stress distribution, compression areas (minimum principal stress) were observed in the buccal cervical region of the PDL in the incisors and canines, as well as in the apical lingual region of their PDLs in S1 (Fig 5A-5D). Among these, the central incisor PDL exhibited the highest compression levels (Fig 5A). In the teeth, compression was concentrated in the cervical region of the roots, particularly on the buccal surface of the incisors, and on the mesial surface of the canine and first premolar, as well as in the attachment of the canine (Fig 5D). In S2, a pattern of compressive stress similar to that observed in S1 was found in the lingual and buccal regions of the incisors’ PDLs and roots. In contrast, compressive stress in the PDL and root of the canine and first premolar was noticeably reduced (Fig 5A, 5B, 5E, 5F). In S3, the canine exhibited an increase in compressive stress on the periodontal ligament and root compared to S2, reaching values similar to those observed in S1 (Fig 5A, 5B). In contrast, the incisors showed the lowest compressive stress on both the PDL and root surfaces among the three simulations (Fig 5A–5H). Interestingly, although the minimum principal stress values for the canine’s PDL and tooth surface were similar in S1 and S3, the distribution of compressive areas differed. In S1, greater compression was observed on the mesial surface of the canine root (Fig 5D), whereas in S3, compression was more prominent on the buccal surface of the root (Fig 5H), suggesting a reduced tendency toward mesial tipping when this tooth is intruded independently.

**Fig 5:**
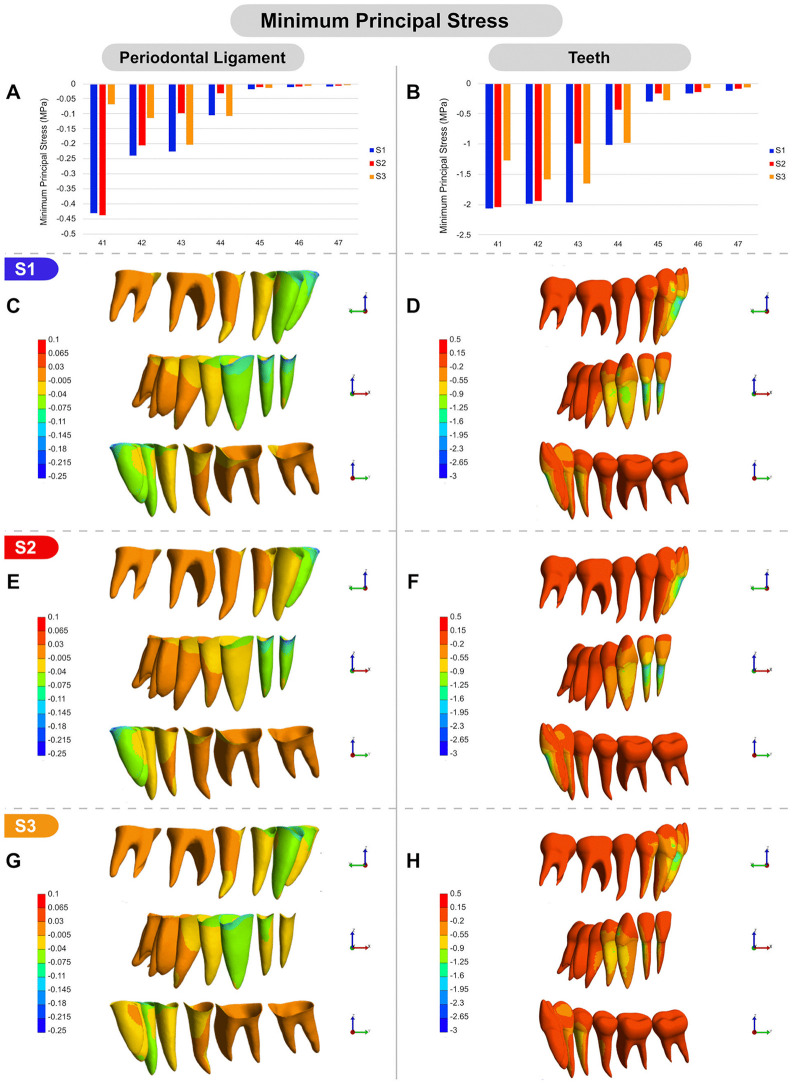
Minimum principal stress (MPa) under S1, S2, and S3 conditions. **(A, B)** Graphical comparison of minimum principal stress values: (A) in the PDL of the lower teeth across S1, S2, and S3; (B) in the lower teeth across S1, S2, and S3 conditions. **(C-H)** Color-mapped visual representations of minimum principal stress distribution: (C, D) under S1 in the PDL and teeth, respectively; (E, F) under S2 in the PDL and teeth, respectively; (G, H) under S3 in the PDL and teeth, respectively. All images (C-H) include buccal, frontal, and lingual views.

When evaluating tension stress (maximum principal stress) under different simulations (Fig 6), S1 exhibited stress concentration in the cervical/mesial and apical/distal regions of the incisors’ PDL, while the canine displayed the opposite pattern. Overall, the apical/lingual portion of the PDL in these teeth experienced lower tension levels (Fig 6C, 6D). In the teeth, the highest tension stress concentration was located in the cervical/lingual region of the incisor roots, as well as in the distal portion of the roots of posterior teeth, with greater intensity in the canine and first premolar and lower intensity in the molars. Additionally, an increased tension area was observed in the attachments of the canine and first premolar (Fig 6D). When comparing S2 to S1, a more homogeneous stress distribution was observed along the PDL of the incisors and canines, suggesting a reduced tendency for mesiodistal tipping of these teeth. Moreover, tension stress in the canine PDL was visibly reduced (Fig 6A, 6E). In the teeth, a distribution pattern similar to S1 was observed, but with lower stress values in the canine, first premolar, and their respective attachments (Fig 6B, 6F). S3 exhibited higher tension stress in the canine PDL, particularly in the mesial and distocervical regions. In the PDL of adjacent teeth, a more uniform stress distribution was observed (Fig 6G). In the teeth, however, a different tension stress pattern emerged, with the highest stress concentration in the cervical/mesial region of the incisor roots. This pattern was also present in posterior teeth, albeit at a much lower intensity (Fig 6H). In the PDL of posterior teeth, tension stress values were close to zero in all simulations.

**Fig 6:**
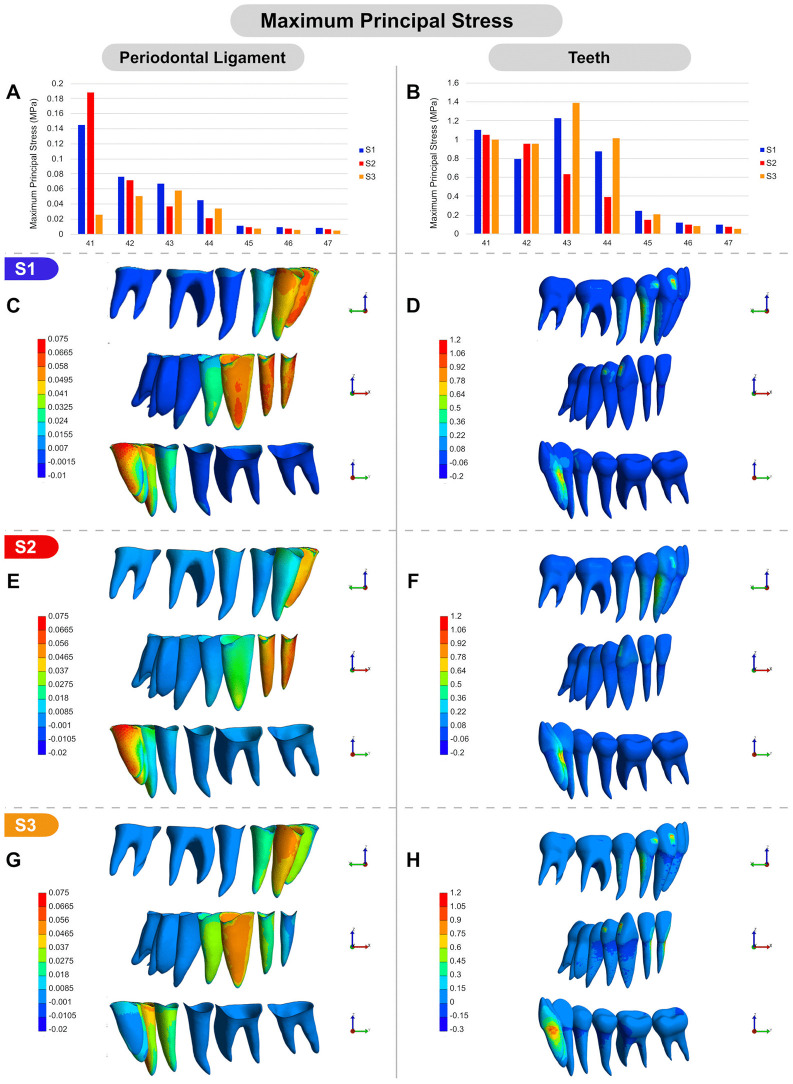
Maximum principal stress (MPa) under S1, S2, and S3 conditions. **(A, B)** Graphical comparison of maximum principal stress values: (A) in the PDL of the lower teeth across S1, S2, and S3; (B) in the lower teeth across S1, S2, and S3 conditions. **(C-H)** Color-mapped visual representations of maximum principal stress distribution: (C, D) under S1 in the PDL and teeth, respectively; (E, F) under S2 in the PDL and teeth, respectively; (G, H) under S3 in the PDL and teeth, respectively. All images (C-H) include buccal, frontal, and lingual views.

#### Movement trend.

Fig 7 illustrates the vector analysis of the overall displacement pattern of the dentition in the three anterior intrusion protocols. In simulations S1 and S2 (Fig 7A, 7B), the central and lateral incisors tended crown intrusion and labial inclination, while the root apex tended to intrude and lingualize. This proclination trend was more pronounced in S2 (Fig 7B) compared to S1 (Fig 7A). In both conditions, the canines showed a tendency for intrusion, labial displacement, and mesial crown inclination, whereas the root apex tended to intrude and incline distally. Although the overall displacement direction of the canine was similar in S1 and S2, its magnitude was noticeably reduced in S2.

**Fig 7:**
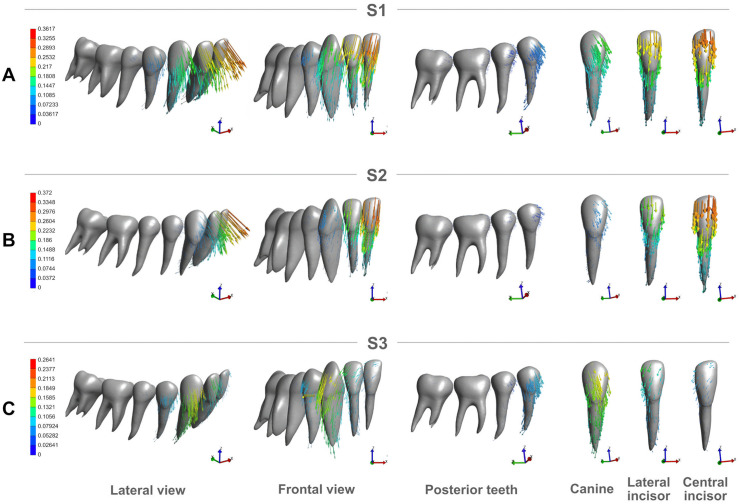
Total movement trend (mm) of the lower teeth under S1, S2, and S3 conditions. **(A)** Lateral and frontal views of the entire lower arch, with displacement vectors indicating the movement trend of the teeth when both incisors and canines are intruded together (S1 condition). Additionally, a lateral view of the posterior teeth segment illustrating the anchorage, alongside individual views of the canine, lateral incisor, and central incisor showing their specific displacement vectors. **(B)** Lateral and frontal views of the entire lower arch, with displacement vectors indicating the movement trend of the teeth when only the incisors are intruded (S2 condition). A lateral view of the posterior teeth segment illustrating the anchorage, alongside individual views of the canine, lateral incisor, and central incisor with their respective displacement vectors. **(C)** Lateral and frontal views of the entire lower arch, with displacement vectors indicating the movement trend of the teeth when only the canines are intruded (S3 condition). A lateral view of the posterior teeth segment illustrating the anchorage, alongside individual views of the canine, lateral incisor, and central incisor showing their specific displacement vectors.

Additionally, in S1, the first premolar tended crown intrusion and mesialization, while the root apex intruded and displaced distally. This pattern was also observed in the second premolar, albeit with lower intensity. The molars displayed a slight mesial crown displacement, with a mild tendency for extrusion of the distal cusps of the second molar. In S2, a similar movement tendency was observed in the anchorage teeth (premolars and molars), but with a significantly lower magnitude.

In S3 (Fig 7C), a distinct movement pattern was observed. The canine exhibited a predominantly intrusive displacement vector, with a slight tendency for labial inclination and mesial crown tipping, while the root underwent a nearly pure intrusion along its long axis. Meanwhile, the incisors demonstrated a minor displacement vector characterized by intrusion, labial movement, and distal crown inclination, with this pattern being more evident in the lateral incisor than in the central incisor. The posterior segment showed a displacement trend closely resembling that observed in S1, but with slightly lower magnitude.

## Discussion

Clear aligner therapy has become an established alternative for orthodontic treatment. However, controlling vertical movements, particularly lower anterior intrusion, remains one of its least predictable aspects [9–14,22–25]. This limitation is mainly attributed to the complex biomechanics of tooth movement with aligners, which involve multiple material and design variables, as well as individual patient factors. Although several clinical studies have evaluated the efficiency of deep bite correction with aligners [14,22,23,25], few have analyzed how different intrusion designs affect stress distribution and force propagation within the system. A deeper understanding of these biomechanical interactions is essential to improve the predictability and effectiveness of COS correction, guiding the development of more efficient digital setups and clinical strategies. This study, using a hemisectioned mandibular model, demonstrated that simultaneous intrusion of canines and incisors (S1) was effective and resulted in the lowest stress distribution on these teeth and surrounding structures. Intrusion of the incisors alone (S2) produced a similar magnitude of incisor displacement but caused minor canine intrusion. When the canine was intruded alone (S3), its displacement and stress distribution were the highest, while the incisors displayed a slight tendency for intrusion with distal crown inclination.

This study used a hemisected mandibular model, applying symmetry boundary conditions in the mid-sagittal plane. Although the existence of inherent anatomical asymmetries that could influence the distribution of biomechanical variables is recognized, the scope of this study focuses on identifying general trends under specific loads, rather than on a patient-specific simulation. As reported by other authors, the symmetry strategy reduces computational cost without losing reliability, a critical factor in models such as the current one, which includes multiple tissues, numerous contact interfaces, and nonlinear materials, such as the periodontal ligament [26,27].

Previous studies have reported variable predictability in deep bite correction and COS leveling with CAT [14,22,23]. Supporting this variability, Shahabuddin et al. (2023) [23] showed that the predictability of deep bite correction with CAT is approximately 33%, with an average intrusive movement of 1.11 mm in the first set of aligners. However, when considering the correction of a 3–4 mm COS, a greater amount of lower anterior intrusion is required. Based on these data, achieving total COS correction would often require multiple refinements and the use of additional features such as bite ramps, skeletal anchorage, or even placement of braces to complete the case, as Sheridan (2004) [24] pointed out. This may be explained by the limited accuracy of lower incisor intrusion with CAT, which has been demonstrated for both manufactured [9–13] and in-house aligners [25]. In individuals with normal occlusion, the average COS depth is approximately 2.0 ± 0.8 mm [1]. Evidence from a recent systematic review and meta-analysis indicates that Class II malocclusions generally present greater COS depth compared with Class I, confirming a consistent tendency for increased curvature in these cases [26].

Finite element analysis (FEA) of deep Curve of Spee (COS) correction using clear aligner therapy (CAT) remains relatively scarce in the literature, despite its clinical significance in managing deep overbite and skeletal Class II malocclusions. In such cases, the extrusion of posterior teeth can contribute to flattening the occlusal plane and mitigating excessive proclination of the lower incisors during anterior intrusion, as previously demonstrated by studies on occlusal leveling biomechanics [27,28]. In the present analysis, posterior teeth were modeled as passive anchorage units rather than actively extruded elements, reflecting clinical conditions in which aligner-induced forces are primarily directed to the anterior region while secondary effects naturally propagate posteriorly. This methodological configuration allows controlled evaluation of the anterior intrusion mechanics while acknowledging that posterior extrusion, especially of premolars and molars, may be beneficial when the COS extends beyond the anterior segment [29]. Clinically, however, achieving true vertical intrusion of the lower anterior teeth remains challenging, particularly in patients with Class II skeletal patterns and labially inclined incisors. Intrusion forces in such cases tend to produce combined intrusive and labial tipping movements, increasing the risk of root resorption and periodontal strain if excessive forces are applied. In our simulation, the 0.25 mm activation represented a standardized aligner deformation, not a clinical displacement, consistent with previous studies that employed comparable activation magnitudes to simulate initial aligner engagement and evaluate resulting stress and displacement trends [27,28,29]. These controlled activations enable meaningful comparisons across different intrusion designs while avoiding overestimation of clinical force magnitudes. Future FEA studies integrating simultaneous anterior intrusion and posterior extrusion could further elucidate their combined biomechanical influence on mandibular curvature correction and overall occlusal leveling.

In parallel, recent finite element investigations using fixed orthodontic appliances have further clarified the biomechanical effects of different COS leveling strategies. Yılmaz et al. (2025) [30] demonstrated that increasing the depth of reverse Curve of Spee NiTi archwires and wire dimensions significantly increases lower incisor displacement and periodontal ligament stress. However, important mechanical differences exist between fixed appliances and clear aligner therapy. While fixed appliances deliver predominantly continuous forces through metallic archwires, aligners generate intermittent, removable-force systems, and the magnitude of force delivery is inherently constrained by the elastic and viscoelastic properties of the polymeric material. Unlike metallic wires, excessive intended activation in aligners is not directly translated into higher orthodontic forces, as large deformations may lead to inadequate adaptation or plastic deformation of the appliance rather than proportional force increase. Complementarily, randomized clinical trials with fixed appliances have shown that clinical COS leveling is typically achieved through a combination of lower incisor intrusion and posterior extrusion. Nasrawi et al. (2022) [31] reported effective COS reduction with minimal external apical root resorption and associated incisor intrusion and proclination, while Ba-Hattab et al. (2023) [32] demonstrated transient reductions in pulpal blood flow during both intrusive and extrusive phases of COS leveling using reverse curve mechanics. Although derived from fixed-appliance systems, these biological responses reinforce the importance of controlled force magnitudes, which is inherently addressed in the incremental activation strategy adopted in clear aligner therapy and in the present finite element model.

Consistently, Hasan et al. (2021) [33] demonstrated through 3D finite element analysis that flattening the Curve of Spee (COS) alters mandibular stress distribution, increasing stress concentrations in the mandibular body and condylar regions. These findings highlight the COS’s role in dissipating masticatory forces and suggest that its modification, whether by function or orthodontic treatment, can influence overall mandibular biomechanics, supporting the relevance of evaluating COS behavior through FEA. [33]

FEA has been widely used in orthodontics and has proven reliable for biomechanical research with CAT [27,34–37]. Using this methodology, various orthodontic movements have been analyzed, such as mass retraction [35], distalization [36], expansion [37], and lower incisor intrusion [27,38]. Among these studies, it is clear that different parameters can affect model quality and result accuracy. Mathematical modeling allows simplification to reduce computational cost without compromising accuracy. In this study, the aligner was modeled in the FEA to better represent the clinical scenario, which is essential for evaluating the real initial stress distribution and force propagation during anterior intrusion movements with CAT.

Our three scenarios highlight these biomechanical differences: (1) S1 resulted in effective intrusion of canines and incisors combined with mesial movement of the anchorage teeth; (2) S2 produced a similar magnitude of displacement to S1 in the incisors but showed higher compressive stress in the PDL and reduced canine intrusion; (3) S3 resulted in higher displacement and stress concentration for the canine, while the incisors displayed a slight tendency for intrusion with distal crown tipping. It is important to highlight that the intrusion pattern observed in S1 appears effective for leveling the curve of Spee, as it promotes intrusion combined with proclination of the anterior teeth, for both canines and incisors. When comparing S1 and S2, no significant change was observed in the intrusion trend of the incisors, indicating that staging this movement separately may not add clinical value. Similarly, when comparing S1 with S3, higher canine intrusion was observed in S3; however, this difference may not be clinically relevant, suggesting that staging canine intrusion separately might unnecessarily increase treatment time without meaningful biomechanical benefit.

These observations are consistent with Liu et al. (2018) [29], who explored the force changes associated with different intrusion strategies using in vitro experiments with a force/moment sensor and 3D-printed resin teeth. They tested four types of intrusion: (1) canine intrusion; (2) incisors intrusion; (3) simultaneous intrusion of canine and incisors; and (4) different activation of canines, lateral and central incisors. Their results demonstrated that each intrusion design exerts distinct forces on the incisors, canines, and anchorage teeth, which aligns with our findings. Specifically, similar intrusion forces were observed when incisors were intruded alone or simultaneously with the canine. Canines intruded together with incisors received only a third of the force they received when intruded alone. This result corroborates the fact that staging canine and incisor intrusion might increase the intrusive force in the canine but does not affect overall incisor intrusion performance, which could lead to reduced treatment efficiency.

Li et al. (2023) [27] analyzed stress distribution and movement trends in lower incisors with different IMPA magnitudes during intrusion with CAT. In their study, the canine was part of the anchorage unit, and a 0.2 mm intrusion was applied, showing an extrusive tendency for the canine unaffected by IMPA magnitude. In our simulation, using a 0.25 mm intrusion displacement, we did not observe canine extrusion when the incisors were intruded alone (S2); however, the canine did tend to move mesially. In that study, it was also found that incisors with an IMPA above 110 degrees showed labial root tipping, which could lead to bone dehiscence and periodontal issues. In our study, a single incisor angulation was used, focusing on how different intrusion designs influence lower anterior biomechanics. Anatomical characteristics of the lower anterior segment, such as initial labial inclination, crown length, and labial bone plate thickness, play an important role in the biomechanical response to intrusion forces. Greater incisor proclination or thinner labial alveolar bone can increase the magnitude of labiolingual moments and stress concentration in the cervical and labial regions of the PDL, predisposing to root movement toward the labial plate [27,29]. These factors may help explain the tendency for labial displacement observed in similar finite element analyses of lower anterior intrusion with aligners, reinforcing the influence of tooth morphology and alveolar anatomy on the resulting force system and stress distribution. We observed that the incisors intruded alone (S2) did not present significant changes in their buccal movement trend compared to S1, although the canines in S1 showed greater buccal crown displacement, indicating a possible proclination tendency. No clear apical inclination differences were observed among the simulations.

Although the present model was based on a mandibular anatomy with physiologic alveolar bone thickness, variations in labial bone morphology may influence the clinical response to lower anterior intrusion. In patients with a thin labial cortical plate, intrusive forces combined with labial tipping may increase the risk of periodontal complications. However, the objective of this study was to assess the initial biomechanical trends generated by different anterior intrusion designs under standardized conditions. At this early stage, displacement patterns are primarily governed by aligner activation, attachment configuration, and periodontal ligament behavior. Patient-specific variables such as alveolar bone thickness, initial incisor inclination, and crown–root proportion should be incorporated in future finite element studies to expand the clinical applicability of these findings.

Intrusion of lower anterior teeth requires space [39]. In our finite element model, no occlusal or interproximal contacts were defined, allowing sufficient mechanical freedom for the anterior teeth to move apically under the prescribed intrusion activation. Therefore, no additional “space creation” was necessary for the simulations. It is important to note that, both biologically and computationally, tooth intrusion does not require a pre-existing apical cavity. In vivo, the initial displacement occurs within the periodontal ligament space, followed by bone remodeling that gradually accommodates the root movement. In our model, this condition is realistically represented by the inclusion of a visco-hyperelastic PDL, which allows deformation and stress dissipation consistent with the early phase of tooth movement. As the present study aimed to analyze the initial biomechanical response rather than the full clinical progression of intrusion, this modeling approach is methodologically appropriate and consistent with previous FEA studies [27,29].

Interestingly, a slight intrusion pattern was observed in the canines in S2 and in the incisors in S3, even though intrusion was not programmed for these teeth in those scenarios. This phenomenon may be related to the immediate propagation of force within the aligner when inserted, but further studies are needed to assess how force distribution behaves over time and whether this tendency would change as treatment progresses.

Thus, several key points can be highlighted: (1) Displacement and stress distribution in the canine are greatest when it is intruded alone (S3); however, in this scenario, the incisors show a slight intrusive tendency with distal crown inclination. (2) Intruding the incisors alone (S2) produces similar displacement to S1. (3) Simultaneous intrusion of canines and incisors (S1) appears biomechanically the most efficient and shows the lowest stress values overall. This model provides a useful reference for clinicians and future studies, but clinical trials are needed to validate these findings.

## Conclusion

This study demonstrated that different lower anterior intrusion designs with CAT produce distinct biomechanical responses: simultaneous intrusion of canines and incisors (S1) appears to be the most efficient strategy for leveling the curve of Spee, while staging these movements separately (S2 and S3) does not provide significant advantages and may unnecessarily extend treatment time. The slight intrusion patterns observed in non-target teeth highlight the importance of anchorage control and understanding force propagation within the aligner. These findings emphasize the need for future clinical trials and improved anchorage strategies to ensure that anterior intrusion with aligners becomes a more predictable and effective movement.

## Supporting information

S1 Fig Von mises equivalent strain. Data for plotting the von Mises equivalent strain for Fig 4.
(XLSX)

S2 Fig Minimum principal stress. Data for plotting the Minimum Principal Stress for Fig 5.
(XLSX)

S3 Fig Maximum principal stress. Data for plotting the Maximum Principal Stress for Fig 6.
(XLSX)
